# A change in microsatellite instability caused by cisplatin-based chemotherapy of ovarian cancer

**DOI:** 10.1054/bjoc.2001.2037

**Published:** 2001-09

**Authors:** Y Watanabe, M Koi, H Hemmi, H Hoshai, K Noda

**Affiliations:** Department of Obstetrics and Gynecology, Kinki University School of Medicine, Osaka, Japan; Department of Molecular Biology, Toho University School of Medicine, Tokyo, Japan; Department of Biological Sciences, Brunel Institute of Cancer Genetics and Pharmacogenomics, Brunel University, Middlesex, UK

**Keywords:** microsatellite instability, CDDP-based chemotherapy, acquired resistance, ovarian cancer

## Abstract

To clarify the mechanism of acquired CDDP resistance in ovarian cancer, we compared the microsatellite instability (MSI) by the amplification of 10 microsatellite loci and immunohistochemical detection of hMSH2 and hMLH1 expression between the primary resected tumours and the secondary resected residual tumours after 5 or 6 courses of CDDP-based chemotherapy in the 24 cases of ovarian cancer. Of the 24 primary resected tumours, 9 (37.5%) showed MSI (7 cases of MSI-L, 2 cases of MSI-H), while 15 (72.5%) were microsatellite stable tumours (MSS). The primary tumours also had MSI in the residual tumours after CDDP-based chemotherapy. However, all of the cases with MSS in the primary resected tumours exhibited MSI (2 cases were MSI-L, and 13 cases were MSI-H) in the residual tumours after CDDP-based chemotherapy (*P*< 0.001). Furthermore, 11 (73.3%) of these cases which changed from MSS to MSI also had a change in the expression of hMLH1 from positive to undetectable (*P*< 0.001). Our data suggest that tumour MSI changes during CDDP-based chemotherapy, and that the loss of hMLH1 expression is one of the factors that has the greatest effect on this transformation. © 2001 Cancer Research Campaignhttp://www.bjcancer.com


*British Journal of Cancer* (2001) **85**(7), 1064–1069
© 2001 Cancer Research Campaign
doi: 10.1054/ bjoc.2001.2037, available online at http://www.idealibrary.com on IDEAL

http://www.bjcancer.com

### A change in microsatellite instability caused by cisplatin-based chemotherapy of ovarian cancer

**Y Watanabe[1], M Koi[3], H Hemmi[2], H Hoshai[1] and K Noda[1]**

[1]Department of Obstetrics and Gynecology, Kinki University School of Medicine, Osaka, Japan; [2]Department of Molecular Biology, Toho University School of Medicine, Tokyo, Japan; [3]Department of Biological Sciences, Brunel Institute of Cancer Genetics and Pharmacogenomics, Brunel University, Middlesex, UK

**Summary** To clarify the mechanism of acquired CDDP resistance in ovarian cancer, we compared the microsatellite instability (MSI) by the amplification of 10 microsatellite loci and immunohistochemical detection of hMSH2 and hMLH1 expression between the primary resected tumours and the secondary resected residual tumours after 5 or 6 courses of CDDP-based chemotherapy in the 24 cases of ovarian cancer. Of the 24 primary resected tumours, 9 (37.5%) showed MSI (7 cases of MSI-L, 2 cases of MSI-H), while 15 (72.5%) were microsatellite stable tumours (MSS). The primary tumours also had MSI in the residual tumours after CDDP-based chemotherapy. However, all of the cases with MSS in the primary resected tumours exhibited MSI (2 cases were MSI-L, and 13 cases were MSI-H) in the residual tumours after CDDP-based chemotherapy ($P < 0.001$). Furthermore, 11 (73.3%) of these cases which changed from MSS to MSI also had a change in the expression of hMLH1 from positive to undetectable ($P < 0.001$). Our data suggest that tumour MSI changes during CDDP-based chemotherapy, and that the loss of hMLH1 expression is one of the factors that has the greatest effect on this transformation. © 2001 Cancer Research Campaign http://www.bjcancer.com

**Keywords**: microsatellite instability; CDDP-based chemotherapy; acquired resistance; ovarian cancer

As the efficacy of postoperative chemotherapy for ovarian cancer is closely related to the prognosis of patients, especially those with advanced cancer, an important issue in the therapy for ovarian cancers is to clarify the mechanisms of resistance to anti-cancer agents.

Cis-diamminedichloroplatinum (CDDP) and taxanes currently are 2 of the most effective anti-cancer agents for ovarian cancer. Currently, chemotherapy regimens for epithelial ovarian cancer have been changed by the results of several clinical trials (Omura et al, 1989; McGuire et al, 1996). However, platinum agents, such as CDDP and carboplatin, still are used as a key-drug in regimens for ovarian cancer. Therefore, understanding the mechanisms of resistance to platinum agents may be helpful for improving the prognosis of patients with advanced ovarian cancer. However, in spite of many molecular biologic studies, such as the investigation of multi-drug resistance (*mdr*) gene expression (Kusaba et al, 1999; Leith et al, 1999), the expression of glutathione-associated enzymes (Nishimura et al, 1996; D'Incalci et al, 1998), and the loss of *p53* function (Bunz et al, 1999; Zheng et al, 1999), there is no clear understanding about the mechanism of the resistance to CDDP acquired during chemotherapy for ovarian cancer, and the optimal management of CDDP-resistant ovarian cancer has yet to be established.

Generally, CDDP-based chemotherapy is administrated for 5 or 6 courses for patients with ovarian cancer as induction chemotherapy, if the residual tumour shows no obvious enlargement or no distant metastases appear during the first few cycles of chemotherapy. Previous large clinical trials revealed that postoperative CDDP-based chemotherapy could achieve a 60% response rate (McGuire et al, 1996) with cyclophosphamide, a 73% response rate with paclitaxel (McGuire et al, 1996), or a 69% response rate (Muggia et al, 2000) with CDDP alone in patients with primary epithelial ovarian cancer. However, the causative factor that prevents improvements in the therapeutic efficacy for advanced ovarian cancer is the acquired resistance to anti-cancer agents including CDDP. The most well-known pattern of this acquisition is that the early phase of chemotherapy fails to achieve a complete remission but causes a partial reduction in the tumour size, and then, with repeated administration of the CDDP-based chemotherapy, the response of the tumour to the anti-cancer agent decreases. Therefore it is essential to elucidate the mechanisms responsible for the decreased responses of the tumour to anti-cancer agents during the course of CDDP-based chemotherapy.

Recent studies have suggested that an abnormality in the DNA mismatch repair system, which has been reported to be the cause of hereditary cancers such as hereditary nonpolyposis colorectal cancer (HNPCC), may be involved in resistance to anti-cancer agents, because the anti-cancer effects of chemotherapy are often dependent upon the generation of DNA damage, which is recognized by the DNA mismatch repair system (Fink et al, 1996; Karran and Hamps, 1996). In addition, recently reported in vitro (Anthoney et al, 1996) and in vivo (Aebi et al, 1996; de las Alas et al, 1997) studies revealed that one of the causative factors of this deficiency is a loss of hMLH1 expression due to the hypermethylation of the promoter for the *hMLH1* gene (Strathdee et al, 1999; Plumb et al, 2000). However, it is still unknown whether the microsatellite instability (MSI) occurred as a result of a deficiency in the DNA mismatch repair system before or after CDDP-based induction chemotherapy in patients with advanced primary epithelial ovarian cancer. Therefore, we conducted a comparative study on MSI and the expression of the DNA mismatch repair-related proteins hMLH1

*Received 26 May 2001*
*Revised*
*Accepted*

*Correspondence to:* Y Watanabe and hMSH2 in primary resected tumours and residual tumours in women requiring a second excision of ovarian cancer following 5 or 6 courses of CDDP-based postoperative induction chemotherapy to manage an incomplete primary resection.

### MATERIALS AND METHODS

### Subjects

The subjects of this study were 24 patients with primary epithelial ovarian cancer in whom the primary operation resulted in a noncurative resection with residual tumours large enough to be measured for the determination of the efficacy of post-operative induction chemotherapy who were treated in our department from 1989 to 1995. Despite a total 5 or 6 courses of postoperative CDDP-based chemotherapy, we performed secondary cytoreductive surgery at 6 to 8 weeks after the final administration of CDDP-based chemotherapy for the cases with a partial response (PR) and no change (NC). Those with a complete response (CR) or progressive disease (PD) did not undergo secondary cytoreductive surgery. Paired tumour samples from these 2 resections were used to assess the effects of CDDP-based chemotherapy on the tumour's microsatellite instability (MSI) and the protein expression of hMSH2 and hMLH1. In this study, we obtained informed consent from all patients prior to examine the specimens.

The clinical stage of each patient was determined according to the classification of the International Federation of Gynecology and Obstetrics (FIGO, 1989). It was stage IIIc in 19 patients and stage IV in 5 patients. The histologic subtypes included serous adenocarcinoma (14 patients), serous papillary adenocarcinoma (3 patients), mucinous adenocarcinoma (1 patient), endometrioid adenocarcinoma (3 patients), clear cell adenocarcinoma (2 patients), and undifferentiated carcinoma (1 patient). The histologic grade was $G_1$ in 4 patients, $G_2$ in 16 patients, and $G_3$ in 4 patients.

The postoperative chemotherapy regimen was CAP (CDDP 50 mg $m^{-2}$, doxorubicin 40 mg $m^{-2}$, cyclophosphamide 300 mg $m^{-2}$), or CP (CDDP 70 mg $m^{-2}$, cyclophosphamide 500 mg). All patients received 5 or 6 courses of this regimen (mean: 5.5 courses), and the mean total dose of CDDP administrated was 427.7 ± 19.1 mg (range: 408–480 mg). The assessment of the tumour's response to chemotherapy was based upon the World Health Organization criteria (1979) and was classified as a partial response (PR) in 11 cases and no change (NC) in 13 cases.

### Preparation of samples

The existence of cancer cells and their histologic subtype and grade were confirmed using haematoxylin and eosin staining of the samples obtained from tumour excised during the primary surgery or the secondary surgery after chemotherapy. Sample slices 4 $\mu$m in thickness were prepared by microdissection from the paraffin-embedded cancerous and normal tissues. DNA was extracted using DEXPAT™ (Takara, Shiga, Japan) to prepare the DNA templates for polymerase chain reaction (PCR).

### Assay for MSI

As primers for the assay for MSI we used 10 microsatellite loci which included BAT25, BAT26, BAT40, D2S123, D3S1612, D5S346, D11S904, D17S795, D18S58, and TP53 (MAPPAIRS™; Research Genetics, Huntsville, AL). Four loci (BAT25, BAT26, D2S123, and D5S346) were recommended for analysing MSI, and 2 loci (BAT40 and D18S58) were recommended as alternative loci as published in the International Guidelines for the Evaluation of MSI in Colorectal Cancer (Boland et al, 1998). We selected 4 more microsatellite loci including D3S1612 (hMLH1) (Hass et al, 1999), D11S904 (Hickey et al, 1999) (chromosome 11p), D17S795 (Pieretti et al, 1995) (chromosome 17q), and TP53 (p53) for the assay of MSI. The forward primer was 5′-end-labelled with [$\gamma^{32}$P]-ATP (Amersham Pharmasia Biotech, Tokyo, Japan) using $T_4$-polynucleotide kinase (Promega, Madison, WI), and was used together with an unlabelled reverse primer.

For the PCR amplification of the microsatellite loci, 1 $\mu$l of each extracted DNA sample was used per 5 $\mu$l reaction mixture containing 1 × PCR buffer (Promega), 2 mM $MgCl_2$, 250 $\mu$M of a dNTP mixture (Promega), 10 pmol each of the unlabelled reverse primer and labelled forward primer, 0.25 unit of Taq DNA polymerase (Promega), and double distilled water. The PCR amplification was performed in a thermal cycler (Perkin-Elmer Cetus, Emeryville, CA) with the following cycling conditions: 2 min at 96˚C followed by 35 cycles of 94˚C for 45 s, 57˚C for 45 s and 72˚C for 60 s, for BAT25, BAT26, D2S123, D11S904, D17S795, and TP53; and 10 min at 95˚C followed by 35 cycles of 94˚C for 30 s, 55˚C for 60 s and 72˚C for 60 s, for BAT40, D3S1612, D5S346, and D18S58. After the final cycle, 7 min at 72˚C was added for a final elongation step.

After PCR amplification, the products were denatured at 94˚C for 5 min, and analysed on 6% polyacrylamide gel (Bio-Rad, Hercules, CA) containing 7 M urea (Wako Pure Chemical, Osaka, Japan). After electrophoresis, the MSI and the loss of heterozygosity (LOH) were confirmed using a Bio-Imaging Analyzer (BAS 1000; FUJIX, Tokyo, Japan) by comparing the microsatellite loci of normal tissue and those of the cancerous tissue. LOH was defined as > 50% visible reduction of the band intensity of one allele of the tumour sample compared with the corresponding normal tissue sample (Pieretti et al, 1995).

### Definition of the MSI

The cases in which there were 7 or more informative loci out of a total of 10 loci examined were used to judge MSI. If an allelic shift between the normal DNA and the cancerous DNA was found in at least one of the products of each of the 10 microsatellite loci examined, it was scored as an MSI tumour. If the MSI was confirmed in 1 or 2 microsatellite loci (<30%), it was scored as MSI-L, while if MSI was confirmed in 3 or more microsatellite loci (≥30%), it was scored as MSI-H according to the criteria of a prior report (Boland et al, 1998).

### Immunohistochemical staining

The same paraffin-embedded cancer tissues as those used for the MSI analysis were sectioned into 4-$\mu$m-thick slices and examined for the expression of hMSH2 and hMLH1 by immunohistochemical staining using anti-hMSH2 and hMLH1 antibodies and a VECTASTAIN ABC kit (Vector Laboratories, Burlingame, CA). For the staining of hMSH2 and hMLH1, we used anti-hMSH2 (Santa Cruz Biotechnology, Santa Cruz, CA) and anti-hMLH1 (Santa Cruz Biotechnology). To confirm the positivity for hMSH2 and hMLH1 expression, staining without either of the primary antibodies served as a negative control. Two slides in each sample

© 2001 Cancer Research Campaign 

were immunostained in separate runs, and were scored by an independent pathologist who was completely blinded to treatment status and patients name. In scoring the immunostained slides, the stain intensity of cancer cells was graded: no staining, weakly positive, and strongly positive, and the percentage of cancer cells that stained positively was also graded: 0–20%, 20–80% and 80–100%. If no staining or weakly positive staining were observed, and the percentage of stained cells were less than 20%, we decided that the expression of hMLH1 or hMSH2 was scored undetectable by the immunohistochemical staining.

### Statistical analysis

We used an exact probability test and signed rank test for statistical analysis. A $P$ value less than 0.05 was regarded as statistically significant.

### RESULTS

### MSI and expression of hMSH2, hMLH1 protein in the primary resected tumours

MSI was detected in 37.5% (9/24) of the primary resected tumours before CDDP treatment while 15 cases were judged as MSS. 5 cases showed MSI in only 1 microsatellite loci (2 cases had MSI in D5S346, 2 cases had MSI in BAT26, and 1 case had MSI in D2S123), 2 cases had MSI in 2 microsatellite loci (1 case had MSI in BAT25 and D18S58, 1 case had MSI in BAT40 and D2S123), 1 case had MSI in 3 microsatellite loci (BAT26, BAT40 and D18S58), and 1 case had MSI in 4 microsatellite loci (BAT25, BAT26, D3S1612 and TP53). Of the tumours which were initially positive for MSI, 77.8% (7/9) were scored as MSI-L and 22.2% (2/9) as MSI-H.

Concerning the expression of hMSH2 and hMLH1 in the primary resected tumours, of the 9 primary resected tumours that were positive for MSI, 4 (44.4%) were undetectable for hMSH2 expression, 3 (33.3%) were undetectable for hMLH1 expression, and 2 (22.2%) were positive for both hMSH2 and hMLH1 expression. The 15 primary resected tumours that were MSS were all positive for both hMSH2 and hMLH1 expression (Table 1). Moreover, LOH was confirmed in 8 (33.3%) of the primary resected tumours. The most frequent LOH loci was TP53 (6/8; 75%), and the histologic subtype of the TP53 LOH cases were serous adenocarcinoma or serous papillary adenocarcinoma. Moreover, none of the LOH cases exhibited a change in the LOH loci or the number of LOH loci in the secondary resected residual tumors after CDDP-based chemotherapy.

### MSI and expression of hMSH2, hMLH1 protein in the secondary resected residual tumours after CDDP-based chemotherapy

All the tumours which were initially positive for MSI were also positive for MSI after CDDP-based chemotherapy. One case had an increase in the number of MSI loci, and 2 initially positive hMLH1 cases became undetectable for hMLH1 expression (Table 2). Moreover, to our surprise, we found that 15 of the 15 (100%) primary tumours which were negative for MSI become positive for MSI ($P$ < 0.001) after CDDP-based chemotherapy (2 cases changed to MSI-L, and 13 cases changed to MSI-H). In addition, there was a significant difference ($P$ < 0.001) in the mean number of loci with MSI during CDDP-based chemotherapy (before, 0.0 ± 0.0; after, 3.9 ± 1.4). Furthermore, the change in the expression of the hMLH1 protein to undetectable in 10 of the 15 (66.7%) secondary resected tumours after CDDP-based chemotherapy was statistically significant ($P$ < 0.001) (Table 3).

**Table 1** Clinicopathologic characteristics of the study subjects with primary ovarian cancer

| | |
|---|---|
| Mean age (range) | 47.9 ± 5.8 (35 – 59) |
| Stage | |
| IIIc | 19 |
| IV | 5 |
| Histologic subtype | |
| Serous adenocarcinoma | 14 |
| Serous papillary adenocarcinoma | 3 |
| Endometrioid adenocarcinoma | 3 |
| Clear cell adenocarcinoma | 1 |
| Mucinous adenocarcinoma | 1 |
| Undifferentiated adenocarcinoma | 1 |
| Histologic grade | |
| $G_1$ | 3 |
| $G_2$ | 16 |
| $G_3$ | 5 |
| Chemotherapy regimens | |
| CAP | 18 |
| CP | 6 |
| Mean treatment courses (range) | 5.5 ± 0.5 (5 – 6) |
| Mean administered dose of CDDP (range) | 427.7 mg ± 19.1 mg (408 – 480 mg) |
| Therapeutic effect | |
| PR | 11 |
| NC | 13 |

Stage was according to FIGO 1989 classification. CAP: cisplatin (CDDP) 50 mg $m^{-2}$ + doxorubicin 40 mg $m^{-2}$ + cyclophosphamide 300 mg $m^{-2}$, CP: cisplatin 70 mg $m^{-2}$ + cycrophosphamide 500 mg. Therapeutic effect was graded using WHO criteria.

*© 2001 Cancer Research Campaign*

**Table 2** Microsatellite status and the expression of hMSH2, hMLH1 in the residual tumours of microsatellite stable primary ovarian carcinoma after CDDP-based chemotherapy

| Case no | Examined microsatellite loci | | | | | | | | | | hMSH2 expression | hMLH1 expression |
|---|---|---|---|---|---|---|---|---|---|---|---|---|
| | BAT25 | BAT26 | BAT40 | D2S123 | D3S1612 | D5S346 | D11S904 | D17S794 | D18S58 | TP53 | | |
| 1 | ▲ | ▲ | ▲ | O | O | ▲ | O | ▲ | O | ▲ | + | N |
| 2 | O | O | ▲ | O | O | O | O | O | O | ▲ | + | N |
| 3 | ▲ | ▲ | O | O | O | ▲ | × | O | LOH | O | + | N |
| 4 | O | ▲ | ▲ | O | ▲ | ▲ | LOH | O | × | LOH | + | N |
| 5 | O | O | ▲ | O | O | ▲ | ▲ | O | ▲ | ▲ | + | N |
| 6 | ▲ | ▲ | ▲ | O | LOH | ▲ | ▲ | O | ▲ | ▲ | + | N |
| 7 | ▲ | O | O | O | O | × | O | ▲ | O | ▲ | + | + |
| 8 | O | ▲ | O | ▲ | ▲ | O | ▲ | O | O | O | + | + |
| 9 | ▲ | O | O | O | ▲ | O | O | O | O | LOH | + | N |
| 10 | ▲ | ▲ | ▲ | O | O | ▲ | O | O | O | O | + | + |
| 11 | ▲ | ▲ | O | O | × | O | O | ▲ | O | O | + | N |
| 12 | ▲ | O | ▲ | O | ▲ | ▲ | O | O | O | LOH | + | N |
| 13 | O | ▲ | ▲ | O | O | ▲ | O | O | O | O | + | N |
| 14 | ▲ | O | O | ▲ | O | ▲ | O | O | O | ▲ | + | + |
| 15 | O | ▲ | O | O | ▲ | ▲ | O | O | × | ▲ | + | + |

O = negative for allelic shift; ▲ = allelic shift has changed from negative to positive; × = not amplified; LOH = loss of heterozygosity; + = positive expression; N = expression has changed from positive to undetectable.

**Table 3** Microsatellite status and the expression of hMSH2, hMLH1 in the residual tumours of microsatellite unstable primary ovarian carcinoma after CDDP-based chemotherapy

| Case no | Examined microsatellite loci | | | | | | | | | | hMSH2 expression | hMLH1 expression |
|---|---|---|---|---|---|---|---|---|---|---|---|---|
| | BAT25 | BAT26 | BAT40 | D2S123 | D3S1612 | D5S346 | D11S904 | D17S794 | D18S58 | TP53 | | |
| 16 | O | ● | O | O | O | ▲ | O | O | O | LOH | + | N |
| 17 | ● | ● | O | O | ● | ● | O | O | O | LOH | – | N |
| 18 | O | ● | O | O | O | O | LOH | O | O | O | – | + |
| 19 | O | O | O | O | O | ● | O | O | × | LOH | + | – |
| 20 | O | ● | ● | O | O | O | O | O | ● | O | + | – |
| 21 | O | O | O | ● | O | O | O | O | O | O | + | – |
| 22 | × | O | O | O | O | ● | O | O | O | O | – | + |
| 23 | ● | O | × | O | O | O | O | O | ● | O | – | + |
| 24 | O | O | ● | ● | O | O | O | O | O | × | + | + |

O = negative for allelic shift; ● = positive for allelic shift; ▲ = allelic shift has changed from negative to positive; × = not amplified; LOH = loss of heterozygosity; + = positive expression; – = undetectable; N = expression has changed from positive to undetectable.

### DISCUSSION

In primary epithelial ovarian cancer, the percentage of tumours positive for MSI has been reported to be no more than 12% (Arzimanoglou et al, 1996) to 26% (Codegoni et al, 1999), and even in ovarian cancers without MSI, it is uncommon to experience little or no tumour reduction with CDDP treatment. Therefore, it is difficult to predict a tumour's resistance to CDDP solely by the presence or absence of MSI in the primary tumour. An important issue in resistance to CDDP-based chemotherapy is the acquired resistance due to genetic mutations in cancer cells during the course of chemotherapy. This obviously would lower the efficacy of the chemotherapy against ovarian cancer, and remains a serious obstacle for improving the prognosis of patients with ovarian cancer. Therefore, it is important to elucidate the mechanisms involved in the changes in the characteristics of cancer cells caused by CDDP-based chemotherapy. The present study revealed that of the 9 patients whose primary tumour samples were positive for MSI, only 1 achieved a PR with a 52% reduction of the residual tumour, while the other 8 cases had a reduction of less than 50% or even tumour growth. Furthermore, in the comparative study of primary resected tumours and secondary resected residual tumours, we found that all of the MSS tumours changed to MSI tumours during CDDP-based chemotherapy, and 87% of these MSS tumours which had a change in their microsatellite status became MSI-H tumours. In the present study we did not identify the mechanism responsible for the loss of hMLH1 expression, but the possibility that changes in *hMLH1* gene expression could occur during CDDP-based treatment is strongly suggested by our results since all of the residual tumours become positive for MSI. In these tumours, the expression of hMSH2 remained stable while the expression of hMLH1 became undetectable in the 67% of the tumours after CDDP-based treatment. Furthermore, even in the patients whose resected primary tumour was positive for MSI, 2 of the residual tumours also become undetectable for hMLH1 expression after CDDP-based treatment. These data suggest that CDDP-based treatment does not have an effect on the expression of hMSH2 but it does affect hMLH1, and this mechanism may be the major cause of the differences in MSI between a primary resected tumour and the residual tumour during CDDP-based treatment. Fink et al (1998) examined 38 paired tumour samples from patients with ovarian cancer before and after CDDP or carboplatin-based chemotherapy, and reported that 66% of the cases demonstrated a reduction of

*© 2001 Cancer Research Campaign*

hMLH1 expression after at least 3 cycles of platinum-based chemotherapy. Mackay et al (2000) also analysed the expression of MLH1 and p53 in 29 paired tumour samples from patients with node-positive breast cancer before and after neoadjuvant chemotherapy that included doxorubicin, epirubicin or CDDP. They reported that the expression of MLH1 significantly decreased after chemotherapy both in terms of the percentage of MLH1-positive cells as well as the staining intensity of the positive cancer cells compared with the specimens obtained before chemotherapy. On the other hand, the expression of p53 did not change after the chemotherapy. Moreover, it was found that the reduction of MLH1 expression after chemotherapy was correlated significantly with a poorer disease-free survival of the patients. They concluded that MLH1-staining status is one of the clinicopathologic prognostic factors in patients with node-positive breast cancer. Samimi et al (2000) also analysed the expression of hMLH1 and hMSH2 in paired ovarian tumour sections from 54 patients with ovarian cancer before and after platinum-based neoadjuvant chemotherapy, and reported that both the expression of hMLH1 and hMSH2 staining were decreased significantly after platinum-based chemotherapy. However, they concluded that immunohistochemical staining for either hMLH1 or hMSH2 was not highly predictive of drug sensitivity as measured by response or survival in patients with ovarian cancer while it was unclear what the mean administered doses of CDDP, carboplatin, doxorubicin or epirubicin were, and the microsatellite status of the primary tumours were, these 2 recent studies concluded that the expression of hMLH1 is reduced after chemotherapy, although they reached conflicting conclusions regarding the role of hMLH1 reduction on the patient's prognosis. Unexpectedly, our present results regarding the expression of hMLH1 and the hMSH2 after CDDP-based chemotherapy are somewhat contrary to the results of Samimi et al (2000). All of our ovarian cancer patients received 5 or 6 courses (mean: 5.5 courses) of CDDP-based chemotherapy, with a total CDDP dose of at least 400 mg (mean: 427.7 mg) for induction chemotherapy for patients with ovarian cancer. Two possibilities regarding the expression of hMLH1 and the hMSH2 after chemotherapy with platinum or alkylating agents may explain these conflicting results: CDDP-based chemotherapy affects both the expression of hMLH1 and hMSH2 during the early treatment courses, because the crosslinking of the DNA strands by the platinum therapy occurs randomly in each DNA strand. However, as the treatment continues, only hMLH1 remains inactive while the expression of hMSH2 recovers. Another possibility is that CDDP-based chemotherapy could affect the expression of hMLH1 and the hMSH2 in both MSS and MSI tumours. However, the effect to hMLH1 expression by CDDP-based chemotherapy will be more frequently observed in the tumours with normal DNA mismatch repair systems.

5 or 6 courses of CDDP-based chemotherapy are standard for patients with ovarian cancer if the tumour does not progress. Loss of sensitivity to the CDDP-based chemotherapy generally occurs after 3 or 4 courses of treatment, and the decision regarding second-line chemotherapy for the relapsed ovarian tumour after CDDP-based treatment depends on whether the relapsed tumour is clinically resistant or sensitive to CDDP-based chemotherapy, was determined by the platinum-free interval. The response rates of recurrent tumours after platinum-based chemotherapy to further platinum-based therapy depends on whether the relapse occurs within or after 6 months from the final administration of the platinum therapy (Ozols, 1997). Therefore, this suggests that CDDP-based chemotherapy may not cause a permanent change in the genetic characteristics on the residual tumour, and that perhaps the acquired resistance of the residual tumour might occur during the late phase of the CDDP-based chemotherapy. We suspect that the reduction of hMLH1 or hMSH2 expression during the early phase of the CDDP-based chemotherapy occurred as a defence mechanism of the cells against DNA-toxic agents, but that the reduction of hMLH1 expression after 5 to 6 courses of therapy might be the final result of acquired resistance to CDDP-based chemotherapy. This reduction in hMLH1 expression might have caused the MSI of the residual tumour after CDDP-based chemotherapy. Perhaps this acquired resistance develops when a residual tumour has long-term, decreased expression of hMLH1 which then affects the status of MSI, since our results were observed in patients who did not achieve a complete remission with CDDP-based chemotherapy.

A recent study (Allen et al, 2000) has reported that MSI is not a frequent event in ovarian cancer whereas LOH is found frequently, and this result suggests that LOH might be more important in the carcinogenesis of ovarian cancer. Our present study only evaluated patients who had primary surgery and then had another operation for the resection of their residual tumours after CDDP-based treatment; the incidence of MSI (37.5%) in these patients was slightly higher than that of LOH (29.2%). Furthermore, all LOH cases were serous-type adenocarcinoma, and no LOH cases were found among those with endometrioid adenocarcinoma, mucinous adenocarcinoma, or clear cell adenocarcinoma, while MSI was found in 5 cases (55.6%) of serous-type adenocarcinoma, 2 cases (22.2%) of endometrioid adenocarcinoma, and 1 case each of mucinous adenocarcinoma and clear cell adenocarcinoma based upon the 10 microsatellite loci examined. These data suggest that the microsatellite loci examined in this study were more suitable for determining MSI than LOH in sporadic-type ovarian cancers. Moreover, the LOH cases had no change either in the number of LOH loci or the location of LOH on the chromosomes, while the MSI status changed in all of the MSS tumours. Our results suggest that the CDDP-based treatment affected only a tumours MSI and not its LOH, at least for patients with relatively poor prognosis tumours.

If a resected primary ovarian cancer is positive for MSI or MSI is acquired during CDDP-based treatment, anti-cancer agents which are effective in the presence of a deficiency in DNA mismatch repair should be used in the second-line chemotherapy regimens. Although a screening test for various anti-cancer agents using a DNA mismatch repair deficient cell line has been reported and may help in the selection of anti-cancer agents for tumours which have genetic instability (Fink et al, 1998), it seems likely that it will be necessary to develop new anti-cancer agents that are more effective against DNA mismatch repair deficient cells to improve the prognosis of patients with advanced ovarian cancer. The results of our present study also might provide the rationale for gene therapy for advanced ovarian cancer by introducing a functional *hMLH1* gene into residual tumours after chemotherapy to maintain sensitivity to CDDP.

### REFERENCES

Aebi S, Kurdi-Haider B, Howell SB et al (1996) Loss of DNA mismatch repair in acquired resistance to cisplatin. *Cancer Res* **56**(13): 3087–3090

Allen HJ, DiCioccio RA, Hohmann P et al (2000) Microsatellite instability in ovarian, other pelvic carcinomas. *Cancer Genet Cytogenet* **117**(2): 163–166

*© 2001 Cancer Research Campaign*

Anthoney DA, Mcllwrath AJ, Gallagher WM, Edlin AR and Brown R (1996) Microsatellite instability, apoptosis, loss of p53 function in drug-resistant tumor cells. *Cancer Res* **56**(6): 1374–1381

Arzimanoglou II, Lallas T, Gilbert F et al (1996) Microsatellite instability differences between familial and sporadic ovarian cancers. *Carcinogenesis* **17**(9): 1799–1804

Boland CR, Thibodeau SN, Hamilton SR et al (1998) A national cancer institute workshop on microsatellite instability for cancer detection and familial predisposition: Development of international criteria for the determination of microsatellite instability in colorectal cancer. *Cancer Res* **58**(15): 5248–5257

Bunz F, Hwang PM, Vogelstein B et al (1999) Distribution of p53 in human cancer cells alters the response to therapeutic agents. *J Clin Invest* **104**(3): 263–269

Codegoni AM, Bertoni F, Colella G et al (1999) Microsatellite instability and frameshift mutations in genes involved in cell cycle progression or apoptosis in ovarian cancer. *Oncol Res* **11**(7): 297–301

de las Alas MM, Aebi S, Los G et al (1997) Loss of DNA mismatch repair: effects on the rate of mutation to drug resistance. *J Natl Cancer Inst* **89**(20): 1537–1541

D'Incalci M, Bunfanti M, Fiebig HH et al (1998) The antitumour activity of alkylating agents is not correlated with the levels of glutathione, glutathione transferase and O6 alkylguanine-DNA-alkyltransferase of human tumour xenografts. EORTC SPG and PAMM Groups. *Eur J Cancer* **34**(11): 1749–1755

Fink D, Nebel S, Howell SB et al (1996) The role of DNA mismatch repair in acquired resistance to cisplatin. *Cancer Res* **56**(13): 3087–3090

Fink D, Nebel S, Howell SB et al (1998) The effect of different chemotherapeutic agents on the enrichment of DNA mismatch repair-deficient tumor cells. *Br J Cancer* **77**(5): 703–708

Haas CJ, Diebold J, Hirschmann A et al (1999) Microsatellite analysis in serous tumors of the ovary. *Int J Gynecol Pathol* **18**: 158–162

Hickey KP, Boyle KP, Jepps HM et al (1999) Molecular detection of tumour DNA in serum and peritoneal fluid from ovarian cancer patients. *Br J Cancer* **80**(11): 1803–1808

Karran P, Hampson R (1996) Genomic instability and tolerance to alkylating agents. *Cancer Surv* **28**: 69–85

Kusaba H, Nakayama M, Wada M et al (1999) Association of 5' CpG demethylation and altered chromatine structure in the promoter region with transcriptional activation of the multidrug resistance 1 gene in human cancer cells. *Eur J Biochem* **262**(3): 924–832

Leith CP, Kopeckey KJ, William CL et al (1999) Frequency and clinical significance of the expression of the multidrug resistance proteins MDR/P-glycoprotein, MRP1, and LRP in acute myeloid leukemia: a Southwest Oncology Group Study. *Blood* **94**(3): 1086–1099

Mackay HJ, Cameron D, Rahilly M, Mackean MJ, Paul J, Kaye SB and Brown R (2000) Reduced MLH1 expression in breast tumours after primary chemotherapy predicts disease free survival. *J Clin Oncol* **18**(1): 87–93

Mcguire WP, Hoskins WJ, Brady MF, Kugera PR, Partridge EE, Look KY, Clarke-Pearson DL and Davidson M (1996) Cyclophosphamide and cisplatin compared with paclitaxel and cisplatin in patients with stage III and IV ovarian cancer. *N Engl J Med* **334**: 1–6

Muggia FM, Braly PS, Brady MF, Sutton G, Niemann TH, Lentz SL, Alvarez RD, Kucera PR and Small JM (2000) Phase III randomized study of cisplatin versus paclitaxel in patients with suboptimal stage III or IV ovarian cancer: a gynecologic oncology group study. *J Clin Oncol* **18**(1): 106–115

Nishimura T, Newkirk K, Cullen KJ et al (1996) Immunohistochemical staining for glutathione S-transferase predicts response to platinum-based chemotherapy in head and neck cancer. *Clin Cancer Res* **2**(11): 1859–1865

Omura GA, Bundy BN, Berek JS, Curry S, Delgado G and Mortel R (1989) Randomized trial of cyclophosphamide plus cisplatin with or without doxorubicin in ovarian carcinoma: A gynecologic oncology group study. *J Clin Oncol* **7**(4): 457–465

Ozols RF (1997) Update of the NCCN ovarian cancer practice guidelines. *Oncology* **11**: 95–105

Pieretti M, Cavalier C, Conway PS et al (1995) Genetic alterations distinguish different types of ovarian cancer. *Int J Cancer* **64**: 434–440

Plumb JA, Strathdee G, Sludden J, Kaye SB and Brown R (2000) Reversal of drug resistance in human tumor xenografts by 2'-deoxy-5-azacytidine-induced demethylation of the hMLH1 gene promoter. *Cancer Res* **60**(21): 6039–6044

Samimi G, Fink D, Varki NM, Husain A, Hoskins WJ, Alberts DS and Howell SB (2000) Analysis of MLH1 and MSH2 expression in ovarian cancer before and after platinum drug-based chemotherapy. *Clin Cancer Res* **6**(4): 1415–1421

Strathdee G, Mackean MJ, Brown R et al (1999) A role of methylation of the hMLH1 promoter in loss of hMLH1 expression and drug resistance in ovarian cancer. *Oncogene* **18**: 2335–2341

WHO handbook for Reporting Results of Cancer Treatment (1979) WHO offset publication No.48, World Health Organization, Geneva

Zheng M, Wang H, Yu B et al (1999) The influence of the p53 gene on the in vitro chemosensitivity of colorectal cancer cells. *J Cancer Res Clin Oncol* **125**(6): 357–360

*© 2001 Cancer Research Campaign* 
